# Impairment of Dendrodendritic Inhibition in the Olfactory Bulb of APP/PS1 Mice

**DOI:** 10.3389/fnagi.2019.00002

**Published:** 2019-01-24

**Authors:** Weiyun Li, Shanshan Li, Lianghua Shen, Junbo Wang, Xuewei Wu, Jing Li, Chunlong Tu, Xuesong Ye, Shucai Ling

**Affiliations:** ^1^Institute of Neuroscience and Anatomy, School of Medicine, Zhejiang University, Hangzhou, China; ^2^Department of Clinical Medicine, Zhejiang University City College, Hangzhou, China; ^3^Biosensor National Special Laboratory, Key Laboratory of BME of the Ministry of Education, Zhejiang University, Hangzhou, China

**Keywords:** Alzheimer’s disease, olfactory dysfunction, olfactory bulb, dendrodendritic inhibition, γ oscillations

## Abstract

Olfactory dysfunction is an early event in Alzheimer’s disease (AD). However, the mechanism underlying the AD-related changes in the olfactory bulb (OB) remains unknown. Granule cells (GCs) in the OB regulate the activity of mitral cells (MCs) through reciprocal dendrodendritic synapses, which is crucial for olfactory signal processing and odor discrimination. Nevertheless, the relationships between the morphological and functional changes of dendrodendritic synapses, particularly the local field potentials (LFPs) as a consequence of olfactory disorders in patients with AD have not been investigated. Here, we studied the morphological and functional changes induced by dendrodendritic inhibition in GCs onto MCs in the OB of amyloid precursor protein (APP)/PS1 mice and age-matched control mice during aging, particular, we focused on the effects of olfactory disorder in the dendrodendritic synaptic structures and the LFPs. We found that olfactory disorder was associated with increased amyloid-β (Aβ) deposits in the OB of APP/PS1 mice, and those mice also exhibited abnormal changes in the morphology of GCs and MCs, a decreased density of GC dendritic spines and impairments in the synaptic interface of dendrodendritic synapses between GCs and MCs. In addition, the aberrant enhancements in the γ oscillations and firing rates of MCs in the OB of APP/PS1 mice were recorded by multi-electrode arrays (MEAs). The local application of a GABA_A_R agonist nearly abolished the aberrant increase in γ oscillations in the external plexiform layer (EPL) at advanced stages of AD, whereas a GABA_A_R antagonist aggravated the γ oscillations. Based on our findings, we concluded that the altered morphologies of the synaptic structures of GCs, the dysfunction of reciprocal dendrodendritic synapses between MCs and GCs, and the abnormal γ oscillations in the EPL might contribute to olfactory dysfunction in AD.

## Introduction

Alzheimer’s disease (AD), the most common neurodegenerative disorder, results in severe memory and learning impairments (Wei et al., 2010). Olfactory deficits have been proposed as a marker of AD because reductions in odor detection thresholds, recognition, and identification occur earlier than dementia in many patients (Wesson et al., 2010; Wu et al., 2013). The driving force of AD pathology is hypothesized to be the formation of toxic amyloid-β (Aβ) peptides that are cleaved from amyloid precursor protein (APP), the subsequent development of neurofibrillary tangles (NFTs), and the resulting, cascade of secondary pathologies (Lachén-Montes et al., 2016). Therefore, studies aiming to reveal the relationship between neuropathological Aβ deposition and olfactory dysfunction have the potential to provide information about early AD pathology, and, ultimately, early diagnosis.

Olfaction involves various processes including sensory neuron inputs to the olfactory bulb (OB), decoding in the primary olfactory cortex, and ultimately transmission to downstream neurons in the amygdala, hippocampus, hypothalamus and nucleus accumbens (Wachowiak and Shipley, 2006; Wesson et al., 2010). The OB constitutes the first relay of the olfactory system (Lepousez and Lledo, 2013). In the OB, the excitatory sensory inputs from excitatory sensory neurons to mitral cells (MCs) trigger the release of glutamate from their lateral dendrites onto the dendrites of granule cells (GCs), and this section mediates the transmission of the GABAergic inhibition back to MCs (Isaacson and Strowbridge, 1998; Lepousez and Lledo, 2013). The recurrent and lateral inhibition supported by dendrodendritic reciprocal synapses between the dendrites of MCs’ and GCs’ mediates key roles in sensory processing, such as the gain control and odor selectivity of MC responses, which are crucial for proper odor discrimination (Abraham et al., 2010; Tan et al., 2010). As the largest population of interneurons in the OB, GCs are involved in the synchronization and establishment of the slow temporal firing patterns of MC activity (Schild, 1988; Friedrich and Laurent, 2001; Nusser et al., 2001; Lledo and Lagier, 2006). The function of GCs in inhibiting MCs has been studied using various approaches. However, researchers have not yet determined whether the morphology and function of dendrodendritic reciprocal synapses between GCs and MCs in the OB are impaired in patients with AD.

MCs extend lateral dendrites in the external plexiform layer (EPL) that are contacted by pedunculated, headed spines arising from the distal apical dendrites of GCs, which results in the establishment of the reciprocal dendrodendritic synapses between GCs and MCs (Phillips et al., 1963; Rall et al., 1966; Price and Powell, 1970). Reciprocal dendrodendritic synapses between GCs and MCs are involved in the generation of γ oscillations (40–100 Hz) in the OB (Lagier et al., 2007). As one band of the local field potential (LFP) oscillations in the OB, γ oscillations are induced by odorants and reflect the synchronized spike discharges from principal neurons (Beshel et al., 2007; Lagier et al., 2007; Lepousez and Lledo, 2013; Osinski and Kay, 2016). Notably, γ oscillations are observed in awake and anesthetized animals, as well as in brain slices *in vitro*. However, the role of γ oscillations in sensory coding, particularly in patients with AD, remains unclear.

In the present study, we identified the relationship between Aβ deposits within the olfactory processing network and odor perception in APP/PS1 mice during aging. In addition, we investigated the morphological and functional changes in the reciprocal dendrodendritic synapses by employing a multi-electrode array (MEA) to illustrate the network-level dynamics in response to pharmacological stimulation under excessive Aβ depositions in APP/PS1 mice at sequential age stages. The results showed that Aβ deposition induced morphological and functional changes in the dendrodendritic synapses in the EPL, and thus, contribute to a more in-depth illustration of the mechanism of olfactory disorders in AD.

## Materials and Methods

### Animals

B6/JNju-Tg (APPswe, PSEN1dE9)/Nju (APP/PS1) mice, which coexpress human PS1 encoding the E9 deletion and mouse APP containing humanized APP and Swedish mutations (K594N, M595L), were employed in this study. The mice were bred and maintained within the animal facility at the Laboratory Animal Center of Zhejiang University, Chinese Academy of Sciences. Age-matched nontransgenic littermate, C57BL/6Nju (C57) mice, were used as controls. The animals were housed with three-five same-sex littermates per cage under standard conditions (20–22°C; 40%–60% humidity; 12-h light/dark cycle; and *ad libitum* access to water and food). All animal experiments were carried out in accordance with the National Institutes of Health guidelines for the care and use of laboratory animals (NIH Publication No. 85-23, revised 1996), and the protocols were approved by the Institutional Animal Care and Use Committee of Zhejiang University. We studied 3–4-month-old (mo), 6–7-mo and 9–10-mo APP/PS1 mice and C57 mice to examine the possible contributions of accumulating Aβ deposits on olfaction over time. Both female and male mice were used in all the experiments. The ratio of female and male mice was approximately 1:1. No differences were observed between female and male mice.

### Buried Food Test

A buried food test, which measures how rapidly an overnight-fasted animal locates a small piece of familiar palatable food, was performed as previously published described with minor modifications (Hu et al., 2016). Briefly, at approximately 24 h prior to testing, the 3–4-mo, 6–7-mo and 9–10-mo APP/PS1 and age-matched C57 mice were weighed and subjected to a food-restricted diet. On the testing day, all the mice were habituated to the testing room for 1 h prior to testing, and the mice were then allowed to acclimate to the cage for 5 min before being transferred to an empty clean cage. A small piece (10 mm cube) of the same food that the mouse was fed daily was then randomly placed in a random corner of a clean mice cage with ~3 cm of woodchip bedding. Before the mouse was transferred, a small piece (10-mm cube) of the same food that the mouse was fed daily was placed ~1 cm beneath the bedding in the clean mice cage. The experimental mouse was then placed in the testing cage at a constant distance from the hidden food. The time it takes the mice to find the food was recorded, and whether the food was consumed was also noted. If the mouse failed to find the buried food within 5 min, the test was stopped, and the latency score was recorded as 300 s. Twelve mice from each group were used in the buried food test.

### Fine Olfactory Discrimination Test

The fine olfactory discrimination test was used to measure the olfactory discrimination ability of the mice by associating olfaction with taste aversion. The test was conducted using previously published protocols (Enwere et al., 2004; Zhu et al., 2014). After the buried food test, the same mice were separated into individual cages and deprived of water for 24 h. Each individual mouse was subjected to two stages of testing, a training stage and a testing stage, to obtain each data point. The training experiment was designed to encourage the mice to associate mango smells with palatable drinks and almond smells with bitterness. For the first training stage, a mixture of 10 ml of double-distilled water and 1 ml of mango extract (Mgo) was placed in a sterile 35 × 10-mm dish to allow the mice to habituate to the Mgo smell. The combination of distilled water and Mgo, which served as a reward for response, was designated [+]. The mice were allowed 2 min to find [+]. Thirty seconds after the mouse finished drinking the solution, a fresh [+] solution was provided. In the trials, the amount of Mgo was sequentially increased to 2.5, 4, 5.5, 7 and 8.5 ml. We repeated the last trial five times, and for the sixth trial, we presented the mice with 8.5 ml of almond extract (ALM) with 10 ml of a 1% denatonium benzoate (DB) solution (Sigma-Aldrich, St. Louis, MO, USA). The combination of ALM and DB was designated [−]. The experimental mice found the [−] solution extremely aversive, learned to associate the bitter taste with the smell of ALM because DB is extremely bitter, and subsequently avoided drinking the [−] solutions. An additional four trials were conducted with the [−] solution to ensure that the mice had learned the association between bitter taste and the smell of ALM. In the testing stage, the mouse was presented with two dishes, one of the dishes mainly contained [+], and the other contained [−]. For example, a 60:40 ratio of the odor components in task indicates that the composition ratio of Mgo in distilled water to almond in 1% DB in one dish was 60:40, and that in the other dish was 40:60. Successful discrimination occurred if the mouse drank [+] and did not perform any the following behaviors: (a) chose [−] rather than [+] or (b) chose both [+] and [−] within 30 s. A task was designated a total failure if the mouse tasted the [−] first. The mice that did not choose within 2 min were excluded from the analysis. Ten trials were conducted in each testing stage. In the additional testing sessions, which incorporated decreased contents of Mgo and ALM in [+] and [−], respectively, an appropriate training stage using the new [+] and [−] solutions was included prior to the testing stage.

### Morris Water Maze Test

The Morris water maze (MWM) test, which was conducted according to previously described protocols (Guo et al., 2017), was performed to test the spatial learning and memory of the mice. After experiencing the buried food and fine olfactory discrimination tests, the same mice were subjected to the MWM test. To test their spatial acquisition abilities, all the mice were trained to find the platform and underwent four trials per day for five consecutive days. During the acquisition phase trials, if the mouse was able to escape onto the hidden platform within 90 s it was allowed to stay on the platform for 5 s. If the mouse failed to find the hidden platform within 90 s, it was guided to the platform by the experimenter and allowed to remain on the platform for 15 s to help it remember the platform’s location. The probe trial was performed 24 h after the last acquisition trial to access the retention of the spatial memory. In this phase, the hidden platform was removed, and the mice were allowed to swim for 90 s in the pool. The numbers of platform crossings were recorded using a computerized tracking system.

### Tissue Preparation

After the MWM tests, six randomly selected mice from each group were sacrificed under deep anesthesia with sodium pentobarbital (50 mg/kg) and perfused with 25 ml of 0.01 M phosphate-buffered saline (PBS, pH 7.4) and then with 100 ml of 4% paraformaldehyde in 0.1 M PB solution (pH 7.4). The OBs were removed, postfixed in the same fixative for 2 h and then cryoprotected for 36 h at 4°C in 0.1 M PB containing 30% sucrose. Olfactory sections (25 μm) were cut coronally using a freezing microtome (Leica CM2100, Germany) at −20°C and collected in a cryoprotectant fluid containing 30% ethanediol and 30% glycerinum in 0.01 M PBS. The olfactory sections were then stored at −20°C for immunofluorescence staining and Nissl staining. Six mice from each group that underwent the behavioral tests were anesthetized, perfused transcardially with ice-cold PBS (pH 7.4), and fixed with 0.5% paraformaldehyde. The brain tissues were then submerged in a Golgi-Cox solution containing 5% potassium dichromate, 5% mercuric chloride and 5% potassium chromate in distilled water. A new set of mice from each group were used for electron microscopy, Western blotting and MEA assays.

### Immunofluorescence Staining

The olfactory sections of each group stored at −20°C were washed three times with PBS, blocked with 10% normal goat serum in PBS, and incubated with primary antibodies against Aβ (6E10, Invitrogen, Carlsbad, CA, USA, 1:500) overnight at 4°C. The sections were then rinsed with PBS and incubated with secondary antibodies conjugated to Alexa Fluor 549 (Invitrogen Technologies, 1:500) for 2 h at room temperature. The sections were subsequently rinsed twice with PBS, mounted on gelatin-coated glass slides with fluorescent sealant and cover-slipped. The sections were subsequently observed and imaged with a laser-scanning confocal microscope (Olympus FV1000, Japan). The photomicrographs were saved as TIFs and quantitatively analyzed using ImageJ software.

### Nissl Staining

For Nissl staining, the olfactory sections from each group were rinsed three times with 0.01 M PBS, mounted on gelatin-coated glass slides and naturally dried at room temperature overnight. The sections were defatted by incubation with 75% ethanol at 37°C for 2 h, stained within 0.1% cresyl violet solution for 10 min at room temperature, and rinsed with water. The sections were then sequentially incubated with 70% ethanol (3 s), 80% ethanol (3 s), 90% ethanol (3 s), 95% ethanol (3 s), absolute ethanol I (3 s), absolute ethanol II (5 min), xylene I (10 min) and xylene II (30 min). Permount was added to the slides, and the slides were then covered with coverslips. Images were captured using an Olympus microscope.

### Golgi-Cox Staining

The brain tissues of each group were submerged in Golgi-Cox solution in the dark for 8 days, and the solution was replaced every 2–3 days. The brains were then dehydrated with a 30% sucrose solution, and the OBs were mounted on the vibratome platform and sectioned at 150 μm. The sections were rinsed twice (5 min each) with distilled water to remove any traces of the impregnating solution. The sections were dehydrated by incubation in 50% alcohol for 5 min, incubated with an ammonia solution (3:1, ammonia: distilled water) for 10 min, rinsed twice with distilled water, and incubated with 5% sodium thiosulfate solution for 10 min in the dark. The sections were then rinsed twice with distilled water, sequentially dehydrated in 50% ethanol I (5 min), 50% ethanol II (5 min), 70% ethanol (5 min), 80% ethanol (5 min), 95% ethanol (5 min), 100% ethanol I (5 min), add 100% ethanol II (10 min), cleared with xylene I (10 min) and xylene II (30 min) and mounted on gelatinized slides using Permount. A confocal microscope was then used to obtain the spine density in apical dendrites of GCs. A Z-stack of the optical section was captured using an Olympus FV1000 instrument with an 60× oil-immersion objective lens and a 10× optical zoom. When capturing the fluorescent signals, an excitation wavelength of 559 nm was used, and the light path was adjusted to mirror and receive the reflected light. For the analyses of the dendritic spine density and the neuron maturity, we adopted the criteria detailed in the published literature (Matsuda and Hisatsune, 2017). The dendritic spines of GCs were divided into mature spines (mushroom and thin spines) and immature spines (filopodia and stubby spines) according to their morphology. In this study, all the dendritic spines were categorized and quantified manually as immature or mature.

### Western Blotting

Western blotting was conducted as described previously (Yang et al., 2016). Six OBs were dissected from APP/PS1 mice and age-matched C57 mice at different ages and then lysed using RIPA buffer supplemented with an EDTA-free protease inhibitor cocktail and phosphatase inhibitors. Twenty micrograms of total protein from each group were separated on 10% SDS-PAGE gels and transferred to PVDF membranes. Protein-bound PVDF membranes were incubated with rabbit anti-postsynaptic density 95 (anti-PSD95), β-actin (1:1,000, Cell Signaling Technology, Danvers, MA, USA) synaptophysin and synapsin I antibodies (1:1,000, Sigma, USA) overnight at 4°C. the membranes were then washed with TBST for 15 min, incubated with a horseradish peroxidase-conjugated goat anti-rabbit antibody (1:2,000; Cell Signaling Technology, Danvers, MA, USA) for 2 h, and then washed with TBST. The membranes were subsequently processed for detection using the ECL system. The protein levels were quantified by assessing their optical densities using Quantity One software and are expressed as ratios relative to β-actin.

### Electron Microscopy

Six mice in each group were anesthetized, and their brains were removed. The OB was sliced coronally into 0.2-mm slices using a vibrating slicer and then postfixed with 2.5% glutaraldehyde in 0.1 M sodium cacodylate buffer (pH 7.4) for 12 h. After three washes with 0.1 M PBS (10 min each), the OB slices were exposed to 1% osmium tetroxide for 2 h, washed several times with water, and dehydrated with a gradient series of alcohol solutions (2 × 10 min with 50%, 2 × 10 min with 70%, 2 × 10 min with 90%, and 2 × 10 min with 100%). The sections were subsequently embedded in epon resin, and randomly selected ultrathin sections were stained with uranyl acetate and lead citrate. Three slides per animal and three fields within each granule layer and EPL per slide were randomly selected to quantify the number of synapses and measure the thickness of the PSD. Each field was imaged at 26,500× magnification using a transmission electron microscope (Tecnai G2 F20 S-TWIN, FEI). The number of synapses and the thickness of the PSD in the granule layer and EPL were analyzed in 15 images from each mouse by an experimenter who was blinded to the treatment and genotype using Image Pro Plus 6.0 software.

### MEA Assay

Six mice from each group were deeply anesthetized, and their brains were rapidly excised from their skulls and submerged in ice-cold cutting solution containing 2.34 mM sucrose, 5 mM KCl, 1.25 mM NaH_2_PO_4_, 5 mM MgSO_4_, 26 mM NaHCO_3_, 25 mM glucose, and 1 mM CaCl_2_ for 5 min. Slices were cut from the brain samples using a vibratome (Leica Microsystems, Germany) while the samples were submerged in a chamber filled with ice cold cutting solution. The OB was cut into 250-μm coronal sections, and the tissue slices were allowed to recover in an oxygenated artificial cerebrospinal fluid (ACSF) solution (119 mM NaCl, 2.5 mM KCl, 1.0 mM NaH_2_PO_4_, 26.2 mM NaHCO_3_, 11 mM glucose, 1.3 mM MgSO_4_, and 2.5 mM CaCl_2_) at 34°C for 0.5 h and then incubated at 28°C for at least for 1 h. OB slices were transferred to an MEA chip (60 Square MEA200/50iR-Ti-gr), continuously perfused with oxygenated ACSF, and saturated with 95% O_2_/5% CO_2_ (pH 7.4 and 325 mOsm/kg) at 28°C at a rate of 1 ml/min. All the experiments were conducted through a 6-min recording in ACSF. In addition, control slices that were continuously treated with ACSF after the initial 5 min showed no change in frequencies, sites of initiation, and level of propagation. We treated the OB slices with a GABA_A_ receptor antagonist (bicuculline, 10 μM) and an agonist (muscimol, 100 μM) to assess the synaptic events between GCs and MCs in response to pharmacological stimulation. The drugs were perfused over the slices through gravity at a rate of 3 ml/min. The chemicals were acquired from Tocris Bioscience (Ellisville, MO, USA) and Sigma-Aldrich Canada (Oakville, ON, Canada). The neuronal network activity was recorded using an MEA (MEA2100-System, Reutlingen, Germany) with 60 platinum electrodes, a 50-μm electrode diameter and a 200-μm interelectrode spacing. An OB slice was positioned over the array such that the slice was in close contact with the microelectrode array electrodes throughout the recording. The electrical activity recorded from each channel was digitized at a sampling rate of 25 kHz and acquired using a MEA2100 amplifier (Multi Channel Systems, Reutlingen, Germany). The temperature in the recording chamber was constantly monitored and maintained at 28°C by a heated perfusion cannula (TC02, Reutlingen, Germany).

### Experimental Design and Statistical Analysis

The data were analyzed and performed using GraphPad Prism version 6.0 (GraphPad software). The escape latency data obtained from the MWM test were analyzed by two-way repeated-measures analysis of variance (ANOVA) with Bonferroni post-tests. For the rest of the data, the statistical significance of the differences in the means between two groups was assessed by Mann-Whitney or two-tailed unpaired *t*-tests, as appropriate. A value of *p* < 0.05 was considered to indicate statistical significance. All the data are expressed as the means ± standard errors of the means (SEMs). In all the figures, *p < 0.05, **p < 0.01, and ***p < 0.001.

## Results

### Olfactory Dysfunction Occurs Earlier Than Cognitive Impairment in APP/PS1 Mice

**Figure 1:**
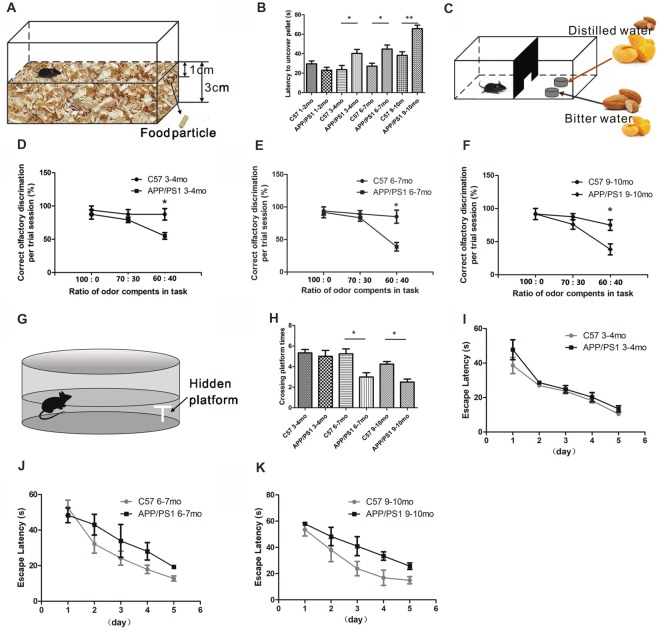
Atypical olfactory and cognitive behaviors of amyloid precursor protein (APP)/PS1 mice. **(A)** Design of the buried food test. **(B)** Quantitative statistical analysis of latency of 3–4-, 6–7 and 9–10-month-old (mo) APP/PS1 and C57 mice to find the hidden food particle. **(C)** Design of the fine odor discrimination test. Mice were deprived of water for 24 h and trained to identify distilled water. In one dish, the distilled water was coupled with an odor mixture of mango and almond, in which mango was the main component. The other dish contained bitter water with an odor mixture in which almond was the main component. Different odor proportions of mango and almond were used in the performance test. **(D–F)** Performance of APP/PS1 and C57 mice at 3–4 **(D)**, 6–7 **(E)**, and 9–10 months of age **(F)**. **(G)** Design of the Morris water maze (MWM) test. **(H)** Quantitative statistics of the crossing platform times of APP/PS1 and C57 mice at 3–4, 6–7 and 9–10 months of age. **(I–K)** Escape latencies of APP/PS1 and C57 mice at 3–4 **(I)**, 6–7 **(J)** and 9–10 months of age **(K)**. The data are presented as the means ± standard errors of the means (SEMs). *p < 0.05; **p < 0.01.

We performed two behavioral tests to assess whether the olfaction function of APP/PS1 mice was altered: the buried food test and the olfactory discrimination test. In the buried food test, the time each individual mouse required to find the hidden food was recorded (Figure 1A). As shown in Figure 1B, the 3–4-mo (*p* < 0.05), 6–7-mo (*p* < 0.05) and 9–10-mo (*p* < 0.01) APP/PS1 mice needed more time to find the hidden food than the age-matched C57 mice, indicating an impairment in their olfactory performance. The same groups of mice that were subjected to the hidden food test were subsequently subjected to the olfactory discrimination test. In the olfactory discrimination test, the mice were acquainted with mango flavor mixed with distilled water and with an almond flavor mixed with bitter water. The olfactory capabilities of the mice were determined based on their ability to distinguish between mixtures containing different fractions of mango and almond smells, which have distinct aromas (Figure 1C). The accuracy of the discrimination between mixtures containing more than 60% of either mango flavor or almond odor based on their smells was comparable. The variable trends in the results from the olfaction discrimination test were consistent with those from the buried food test. Compared with the C57 mice, the 3–4-mo (*p* < 0.05, Figure 1D), 6–7-mo (*p* < 0.05, Figure 1E) and 9–10-mo (*p* < 0.05, Figure 1F) APP/PS1 mice showed decreases in the numbers of correct olfactory discrimination responses per trial, indicating that the level of olfactory discrimination became increasingly impaired as the animals aged. Thus, APP/PS1 mice exhibited olfactory dysfunction at 3–4 months of age.

We then evaluated the hippocampal-dependent spatial learning and memory abilities of APP/PS1 and age-matched C57 mice at sequential age stages using the MWM test (Figure 1G). In this test, the escape latencies of the mice were measured during the 5-day acquisition phase, and the time of platform site crossings was recorded in the probe trial. There were no significant differences in the crossing plate times between the APP/PS1 and age-matched C57 mice at 3–4 months of age (*p* < 0.05, Figure 1H). In addition, no significant differences in escape latency were found in the acquisition phase trials between the APP/PS1 and C57 mice at 3–4 months of age (*F* = 3.883; *p* = 0.0628 > 0.05, Figure 1I), indicating that the 3–4-mo APP/PS1 and control mice have equal abilities to encode and remember the spatial coordinates of the platform. At 6–7 months of age, significantly decreased crossing plate times in probe trial (*p* < 0.05, Figure 1H) and a tendency to require a longer time to find the platform (*F* = 0.2689; *p* = 0.1049 > 0.05, Figure 1J) were observed in the APP/PS1 mice compared with the control mice, indicating that the spatial memory of APP/PS1 mice was impaired. The 9–10-mo APP/PS1 mice needed a longer time to find the platform (*F* = 9.12; *p* = 0.0056 < 0.01, Figure 1K) and exhibited fewer crossings over the former platform site than the control mice (*p* < 0.05, Figure 1H). The above-mentioned results showed that the spatial discrimination learning and memory impairments in APP/PS1 mice started to occur at 6–7 months of age. Based on the results from the behavioral tests, the 3–4-mo APP/PS1 mice suffered olfactory deficits but not cognitive impairments. We thus hypothesized that the 3–4-mo APP/PS1 mice can be considered to be at a relative early stage of AD. In addition, the 9–10-mo APP/PS1 mice suffered olfactory deficits and cognitive impairments and were considered representative of late-stage AD.

### Deposition of Soluble Aβ Aggregates in the Olfactory Bulb

**Figure 2:**
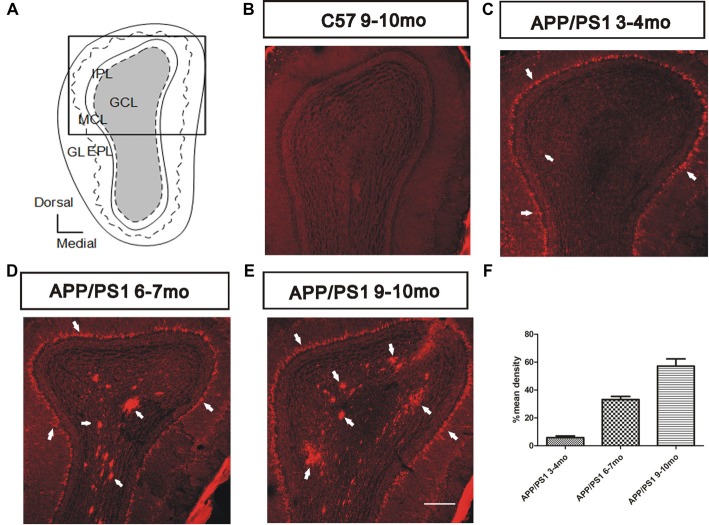
Spatiotemporal pattern of amyloid-β (Aβ) depositions in the olfactory bulb (OB) of APP/PS1 mice. **(A)** Image of the OB coronal plate indicating the bulbar cell layers [glomerular layer (GL), external plexiform layer (EPL), MCL, inner plexiform layer (IPL) and granule cell layer (GCL)]. **(B)** No Aβ was observed in the OB of C57 mice at 9–10 months of age. **(C–E)** Representative images of Aβ staining (white arrows) in the OB of APP/PS1 mice at 3–4, 6–7 and 9–10 months of age. Scale bars = 200 μm in (**E**; applies to **B–E**). **(F)** The mean percentage density of deposited Aβ was elevated in the GCL within the OB cell layers of APP/PS1 mice. The error bars indicate the SEMs.

According to recent evidence, soluble Aβ is strongly correlated with olfactory dysfunction in patients with AD (Wu et al., 2013; Wang et al., 2016). We characterized Aβ deposition in APP/PS1 mice through immunostaining for Aβ using the anti-Aβ antibody 6E10 (1:2,000 diluted) to investigate the spatiotemporal pattern of Aβ aggregates in the OB (Figure 2A). The results showed gradual increases in the amount, degree of aggregation, and spatial distribution of Aβ deposits from the outer to the inner layers in APP/PS1 mice with increasing age and negative staining for Aβ in 9–10-mo C57 mice (Figure 2B). At 3–4 months of age, the MCL developed obvious Aβ deposits (Figure 2C). In addition, Aβ began to accumulate in the GC layer (GCL) and was secreted into the extracellular space (Figure 2C). At 6–7 months of age, the Aβ burden spread throughout the GCL (Figure 2D), and an increased Aβ burden was observed in all layers in 9–10-mo APP/PS1 mice (Figure 2E). In both 6–7-mo and 9–10-mo APP/PS1 mice, Aβ was almost exclusively located within the GCL and MCL in APP/PS1 mice (Figure 2F), and only rare Aβ deposits were observed in the glomerular layer (GL), EPL, or inner plexiform layer (IPL).

### Effect of Aβ Exposure on the Laminar Organization of the OB

**Figure 3:**
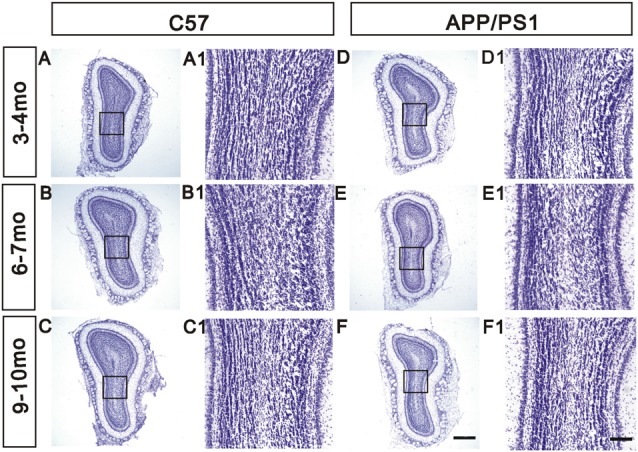
Unchanged laminar organization of the OB in APP/PS1 mice. **(A–F)** No significant change in the laminar organization of the OB was observed between APP/PS1 and age-matched C57 mice at different ages. **(A1–F1)** High-magnification images showing the structure of the GCL. Scale bars = 500 μm in (**F**; applies to **A–F**) and 100 μm in (**F1**; applies to **A1–F1**).

Nissl staining for Nissl bodies was performed to visualize the structure of the OB and thus explore the effects of Aβ depositions on the overall morphology of the OB. A distinct laminar organization, with intact glomeruli and clear layers, was observed in the OB. During aging, the morphology of the OB remained unchanged in APP/PS1 mice compared with the age-matched C57 mice (Figures 3A–F,A1–F1).

### The Dendritic Spine Density of GCs Is Reduced in the OB of APP/PS1 Mice

**Figure 4:**
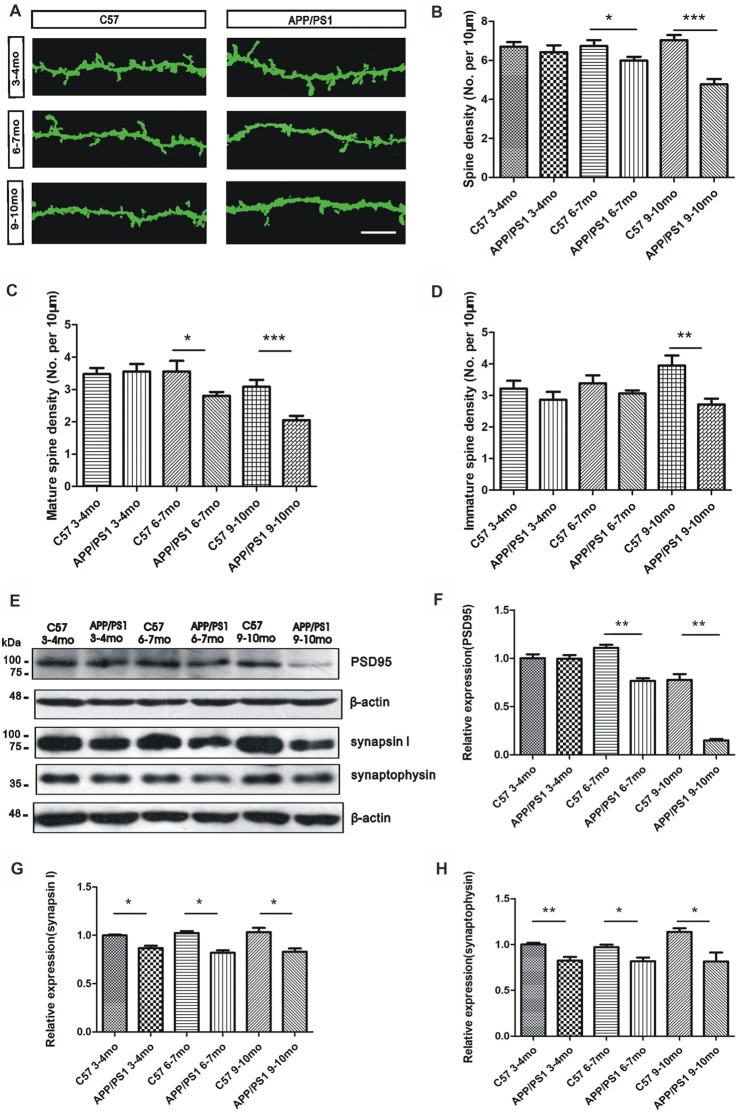
Reduced dendritic spine density of GCs in APP/PS1 mice. **(A)** Representative images of Golgi-stained apical dendritic spines of GCs in APP/PS1 and C57 mice at 3–4, 6–7 and 9–10 months of age. Scale bar = 10 μm. **(B–D)** Quantitative analysis of the dendritic spine density **(B)**, mature spine density** (C)** and immature spine density **(D)** from randomly selected dendritic segments of GCs in APP/PS1 and age-matched C57 mice. **(E–H)** Western blotting assays of postsynaptic density95 (PSD95; **F**), synapsin I **(G)** and synaptophysin **(H)** in the OB; the levels of these proteins were reduced in APP/PS1 mice compared with age-matched C57 mice. The data are expressed as the means ± SEMs. *p < 0.05; **p < 0.01; ***p < 0.001.

We analyzed the dendritic spine density of GCs in the OB through Golgi staining to investigate the mechanisms through which Aβ mediates olfactory deficits in APP/PS1 mice. The morphology of the dendritic spines in GCs in the adult OB is heterogeneous based on the dendrite distributions and dendro-dendritic synaptic partners (Nagayama et al., 2014; McDole et al., 2015). An examination of the dendritic spine density of GCs indicated a qualitatively significant decrease in the APP/PS1 mice compared with the age-matched C57 mice (Figure 4A). The dendritic spine density of GCs in APP/PS1 mice tended to decrease at 3–4 months of age (*p* > 0.05, Figure 4B), but significant decreases were observed in both 6–7-mo (*p* < 0.05, Figure 4B) and 9–10-mo (*p* < 0.001, Figure 4B) APP/PS1 mice compared with their age-matched control mice. The spines of GCs were classified as mature and immature, and confocal observations revealed that the mature spine density was approximately the same in 3–4-mo APP/PS1 and C57 mice (*p* > 0.05, Figure 4C), but significantly impaired in 6–7- and 9–10-mo APP/PS1 mice compared with their age-matched C57 mice (*p* < 0.05, Figure 4C). Compared with that in the age-matched control mice, the immature spine density of GCs in APP/PS1 mice tended to decline at 3–4 and 6–7 months of age (*p* > 0.05, Figure 4D) but was significantly reduced at 9–10 months of age (*p* < 0.01, Figure 4D). We further explored the molecular mechanisms underlying Aβ overexpression-induced olfactory deficits. Specifically, a Western blotting analysis was performed to examine the levels of several synaptic proteins, including a postsynaptic protein (PSD95) and two presynaptic proteins (synaptophysin and synapsin I), and the results revealed that their expression was gradually decreased in APP/PS1 mice compared with C57 mice (Figures 4E–H).

### Amyloid-Beta Overexpression Impairs Synaptic Ultrastructure Parameters

**Figure 5:**
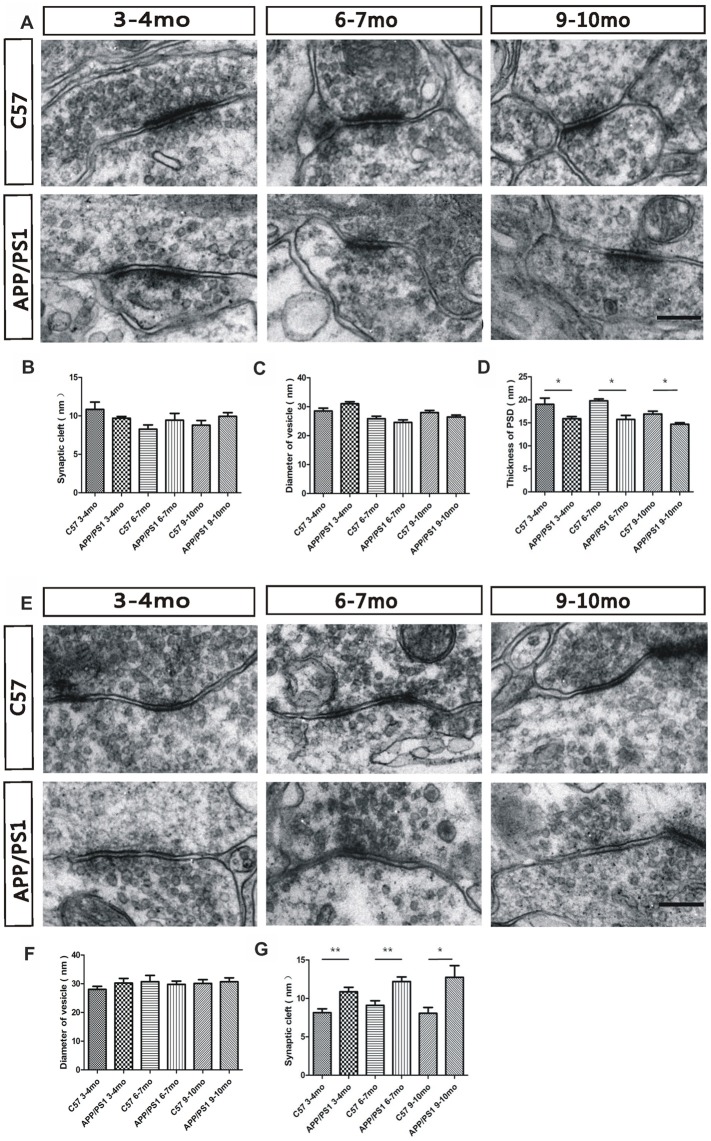
Ultrastructural characterization of dendrodendritic synapses in the EPL. **(A)** The morphology of asymmetric synapses between GC dendrites and mitral cell (MC) dendrites in the EPL of APP/PS1 and C57 mice. **(B,C)** The synaptic clefts and diameter of the vesicles in asymmetric synapses were not significantly different between APP/PS1 and age-matched C57 mice. **(D)** The APP/PS1 mice showed a reduced thickness of the PSD in asymmetric synapses compared with the age-matched C57 mice. **(E)** Morphological structures of symmetric synapses between GC dendrites and MC dendrites in the EPL of APP/PS1 and C57 mice. **(F)** The diameters of the vesicles in symmetric synapses were not significantly different between APP/PS1 and age-matched C57 mice. **(G)** The synaptic clefts were wider in APP/PS1 mice than in age-matched C57 mice. The data are presented the means ± SEMs. The scale bar represents 200 nm. *p < 0.05; **p < 0.01.

An electron microscopy analysis was performed to investigate possible changes in the morphology of the reciprocal dendrodendritic synapses in the EPL. Specifically, we examined various synaptic components, including the minor axis diameter of the synaptic vesicle, the synaptic cleft size, and the thickness of PSD, to demonstrate the distinctive features of reciprocal dendrodendritic synapses. At 3–4 months of age, the synaptic vesicles surrounding the synaptic membrane were transparent and clear in both APP/PS1 and C57 mice (*p* > 0.05, Figure 5A). However, the presynaptic and postsynaptic membrane structures of asymmetric synapses, which are considered excitatory synapses, were damaged. At 6–7 and 9–10 months of age, an unclear and broken membrane structure and blurred boundaries for the synaptic vesicles were observed in APP/PS1 mice. Significant differences in the synaptic cleft (*p* > 0.05, Figure 5B) and the diameters of synaptic vesicles (*p* > 0.05, Figure 5C) were not observed between APP/PS1 and C57 mice. However, compared with the C57 mice, the thickness of the PSD (*p* < 0.05, Figure 5D) in asymmetric synapses was thinner in 3–4-, 6–7-, and 9–10-mo APP/PS1 mice. We subsequently examined the structures of the symmetric synapses in the EPL (Figure 5E), which are also known as inhibitory synapses, and the thickness of the presynaptic membrane was similar to that of the postsynaptic membrane. The integrated structures of synaptic membranes and the approximate diameters of the synaptic vesicles in APP/PS1 mice were similar to those of the C57 mice (*p* > 0.05, Figure 5F). However, the APP/PS1 mice displayed a wider synaptic cleft than the age-matched C57 mice (Figure 5G). These results demonstrate morphological alterations in the reciprocal dendrodendritic synapses in the EPL of APP/PS1 mice, which potentially indicates changes in the function of synapses between GCs and MCs in the presence of excess Aβ depositions.

### Pharmacological Characterization of γ Oscillations in APP/PS1 Mice

**Figure 6:**
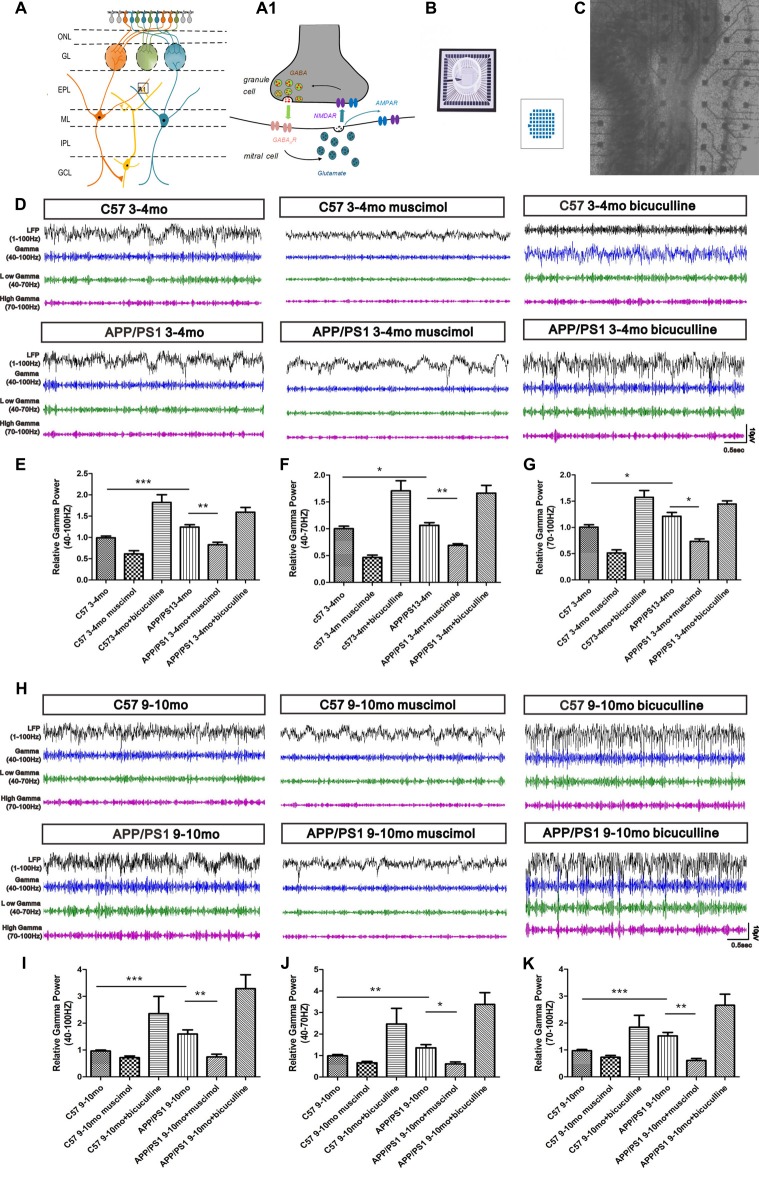
Spontaneous γ oscillations in the EPL of OB slices. **(A)** Schematic showing the locations of MCs and GCs and the dendrodendritic synapses between MC and GC dendrites. Dendrites of MCs receive signals from olfactory sensory neuron termini in the GCL and form synaptic connections with GCs in the EPL. **(A1)** Action potential (AP) propagation in the MC lateral dendrites release glutamate (black vesicles) to activate postsynaptic AMPA and NMDA receptors in the GC dendrites. In turn, NMDA receptors trigger GABA release (red vesicles) and postsynaptic activation of GABA_A_R in MCs. In addition, glutamate released from MCs also activates extrasynaptic glutamate autoreceptors on MC lateral dendrites, resulting in spontaneous excitatory transmission. **(B)** Example of a 60-channel multi-electrode array (MEA). **(C)** Photograph of an OB slice placed on an MEA chip; the subregions of the OB are indicated. **(D)** Representative traces of local field potential (LFP) signals (1–100-Hz bandwidth) and filter traces (γ, low-γ and high-γ) of the EPL in the C57 group, APP/PS1 group, APP/PS1 + muscimol group and APP/PS1 + bicuculline group at 3–4 months of age. **(E–G)** Muscimol weakened the aberrantly increased γ oscillations in 3–4-mo APP/PS1 and C57 mice. Bicuculline enhanced the aberrantly elevated γ oscillations in 3–4-mo APP/PS1 and C57 mice. **(H)** Representative traces of LFP signals (1–100-Hz bandwidth) and filter traces of the EPL in 9–10-mo C57 and APP/PS1 mice. **(I–K)** Muscimol weakened the aberrantly increased γ oscillations in 9–10-mo APP/PS1 and C57 mice, and bicuculline enhanced the aberrantly elevated γ oscillations in 9–10-mo APP/PS1 and C57 mice. The data are presented as the means ± SEMs. *p < 0.05; **p < 0.01; ***p < 0.001.

In the OB slices, the LFPs in the EPL are induced by dendrodendritic excitation/inhibition between GCs and MCs (Figures 6A,A1). The LFP signals in the OB are composed of bursts of γ oscillations (40–100 Hz), which are split into two subbands, the low-γ (40–70 Hz) band and the high-γ (70–100 Hz) band. We investigated the LFPs in the OB through a spontaneous exploration of 3–4-mo and 9–10-mo APP/PS1 and C57 mice using a MEA to assess the effect of Aβ on γ oscillations (Figures 6B,C). The 3–4-mo APP/PS1 mice were in the early phase of olfaction disorder, and the 9–10-mo APP/PS1 mice suffered serious olfaction disorder. The trend in the variations in γ oscillations was similar in the 3–4-mo and 9–10-mo experimental mice and reflected the aberrant synaptic transmission in both the early and late stages of AD.

However, the difference between APP/PS1 and control mice at 9–10 months of age was greater than that at 3–4 months of age. At 3–4 months of age, increased γ, low-γ and high-γ oscillations were observed in the EPL of APP/PS1 mice compared with their age-matched C57 mice (Figures 6D–G). Pharmacological treatments with a GABA_A_R agonist (muscimol) significantly reduced the γ oscillations, and treatment with an antagonist (bicuculline) notably increased the γ oscillations (Figures 6D–G). The γ, low-γ and high-γ oscillations in the EPL of 9–10-mo APP/PS1 mice were substantially enhanced compared with those in their age-matched C57 mice (Figures 6H–K). Treatment with GABA_A_R agonist (muscimol) reduced the γ, low-γ and high-γ oscillations in APP/PS1 mice, whereas the GABA_A_R antagonist (bicuculline) boosted the γ, low-γ and high-γ oscillations (Figures 6H–K).

### Amyloid-Beta Overexpression Induces the Overexcitability of MCs

**Figure 7:**
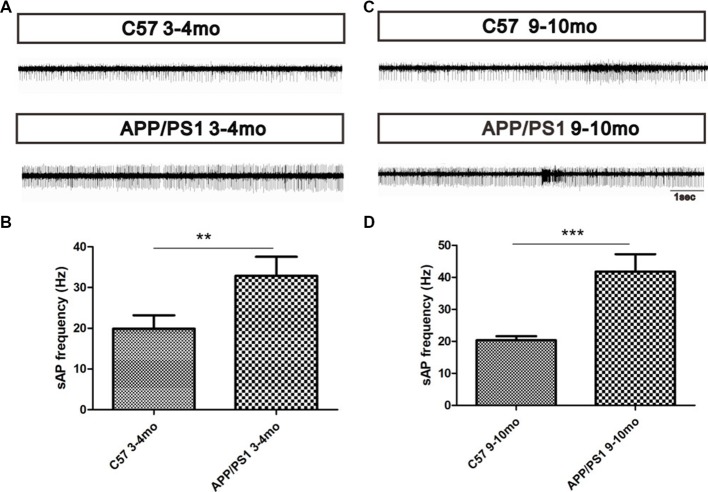
Increased MC spontaneous firings in APP/PS1 mice. **(A)** Spontaneous firing rates of MCs in APP/PS1 and C57 mice at 3–4 months of age. **(B)** Quantitative analysis of spontaneous firing rates of MCs in APP/PS1 and C57 mice at 3–4 months of age. **(C)** Spontaneous firing rates of MCs in APP/PS1 and C57 mice at 9–10 months of age. **(D)** Quantitative analysis of spontaneous firing rates of MCs in APP/PS1 and C57 mice at 9–10 months of age. The data are presented as the means ± SEMs. **p < 0.01; ***p < 0.001.

To explore the effect of Aβ deposition on the spontaneous activity of MCs, a MEA was used to record the MC spontaneous firing rate of spontaneous action potentials (sAPs). The results showed that MCs in APP/PS1 mice at 3–4 (*p* < 0.01, Figures 7A,B) and 9–10 months of age (*p* < 0.001, Figures 7C,D) displayed a relatively higher spontaneous firing rate of sAP than their age-matched control mice (Figures 7A–D), indicating the hyperactivation of MCs in both the early and late stage of AD.

## Discussion

In this study, we investigated the structural and functional changes in the reciprocal dendrodendritic synapses between GCs and MCs in the OB of APP/PS1 mice. During aging, increases in the Aβ deposition in the GCL induced a gradual intensification of olfactory deficits. A reduced dendritic spine density in GCs, decreased protein levels of synapse markers in the OB, and damaged reciprocal dendrodendritic synaptic structures were observed in the APP/PS1 mice. Functionally, disrupted γ oscillations and increased firing rates of MCs were observed in the OB of APP/PS1 mice. The morphological and functional changes in the reciprocal dendrodendritic synapses suggested that the impaired inhibition of MCs from GCs might be a critical mechanism underlying olfactory dysfunction in patients with AD.

As the most common neurodegenerative disorder in the elderly, AD is clinically characterized by progressive declines in memory and learning symptoms, which occur later than olfactory deficit (Djordjevic et al., 2008; Wu et al., 2013; Hu et al., 2017). Olfaction, including the detection threshold, odor identification and odor discrimination, is one of the most important sensations in mammals (Lepousez and Lledo, 2013). AD is a progressive neurodegenerative disease induced by multiple factors, such as apolipoprotein E genetic risk variants, excessive levels of phosphorylated tau protein, immunoinflammatory responses and Aβ deposition, all of which contribute to AD pathogenesis through the Aβ-dependent pathway (Chen et al., 1996; Yu et al., 2014; Moosavi et al., 2015). Most scholars believe that Aβ is the common pathway to AD and is caused by diverse stimuli that play critical roles in the pathogenesis of AD. In addition, it has been hypothesized that Aβ deposits in the olfactory system promote AD pathology in rodents (Wesson et al., 2010, 2011). *In vivo*, excessive Aβ depositions within the hippocampus lead to learning and memory deficits (Kim et al., 2013; Birnbaum et al., 2015; Lei et al., 2016; Wei et al., 2018). Similarly, Aβ depositions in the OB affected olfactory-related behavioral performances in APP/PS1 mice. As the main etiology of AD, the expression and location of Aβ deposits play critical roles in the pathological progression of AD. The degree of Aβ aggregates that accumulate in cognitive cortices through the olfactory pathway during aging is associated with the degree of olfactory dysfunction, ranging from the odor detection threshold to spatial memory (Wu et al., 2013). Based on the results from the olfactory behavioral tests, the APP/PS1 mice displayed progressively aggravated olfactory deficits at 3–4 months of age, when nonfibrillar Aβ depositions were mainly detected within MCs but slightly observed within the GCL. The Aβ depositions in the OB of APP/PS1 mice at 3–4 months of age precedes previous reports of Aβ in the entorhinal cortex and hippocampus, which are involved in learning and memory (Wu et al., 2013; Vasavada et al., 2015; Misiak et al., 2017), and this finding might explain why the 3–4-mo mice suffered olfactory deficits but did not experience memory impairments. The APP/PS1 mice suffered serious olfactory deficits at 6–7 and 9–10 months of age, when the Aβ depositions were mainly accumulated in the GC and MC layers. The GC-mediated regulation of MCs in the OB constitutes the basis for olfactory information processing and transmission processes. Whether the dendritic morphology of GCs and the GC-mediated regulation of MCs in the presence of excessive Aβ deposition were the focus of our research.

In the OB, the functional regulation of GCs is essential for the normal processing of odor information (McDole et al., 2015). In addition, the dendritic morphology of GCs endows the spines with various functional properties (Jiang et al., 2015). Dendritic spines are highly plastic structures that are capable of undergoing adaptive morphological and physiological changes, both during development and in adulthood (Engert and Bonhoeffer, 1999; Tada and Sheng, 2006; Yoshihara et al., 2009; Bosch and Hayashi, 2012; McDole et al., 2015). The dendrite morphology is regulated by many factors, including, PSD95 which can regulate the structure and function of dendritic spines in the brain (Engert and Bonhoeffer, 1999). The neurotransmitter release of synaptic vesicles at nerve terminals involves synaptic vesicle-associated proteins, including synaptophysin and synapsin I, which are related to synaptic transmission and synaptic plasticity in neural networks (Pieribone et al., 1995; Zhang et al., 2016; Chai et al., 2017). Our results showed nonsignificant changes in the dendritic spine density and in the numbers of mature and immature dendritic spines of GCs and decreased protein levels of synaptophysin and synapsin I in 3–4-mo APP/PS1 mice. The results implied that decreased synaptic vesicle-associated protein levels preceded the impairments in the synaptic structure and density. However, the APP/PS1 mice started to show olfactory deficits at 3–4 months of age. We hypothesized that the dendritic spines that form synapses could survive but might have been damaged for a long time. This finding indicated that the impaired synaptic transmission caused by decreased levels of synaptic vesicle-associated proteins, which could be verified by aberrant increases in γ oscillations, might play a key role in abnormal olfactory information integration in the OB and olfactory deficits at the early stage of AD. The 6–7-mo and 9–10-mo APP/PS1 mice showed a gradual accumulation of Aβ depositions within the OB and an aggravated AD pathology, and these effects were accompanied by gradual decrease in the synaptic protein levels and dendritic spine density of GCs, which suggested a decreased number of inhibitory synapses and a weaker inhibitory effect of GCs on MCs in the presence of excessive Aβ depositions. The decreased expression of synaptic-associated proteins and the altered morphology of dendrites of GCs in APP/PS1 mice indicated GC dysfunction in the presence of excess deposited Aβ.

In the OB, GCs establish most of their connections with MCs through ubiquitous dendrodendritic synapses, known as “reciprocal synapses” (Schoppa and Urban, 2003; Shepherd et al., 2007; Bardy et al., 2010), and these reciprocal synapses consist of a presynaptic site of an asymmetrical synapse from MCs to interneurons and a postsynaptic site of a symmetrical synapse that reciprocally connects interneurons to mitral/tufted cells (Isaacson and Strowbridge, 1998). At the early stage of olfactory deficit, pathological changes in the reciprocal synaptic structures in the EPL of APP/PS1 mice were observed, and these changes were followed by more severe lesions with increasing age. At the late stage of AD, the asymmetrical synapses from MCs to interneurons suffered serious damage, such as blurred membrane boundaries and a decreased thickness of the PSD, suggesting aberrant excitatory transmission from MCs to GCs. The integrated structures of synaptic membranes, the approximate diameters of synaptic vesicles, a wider synaptic cleft in the symmetrical synapses, and the decreased dendritic spine density of GCs observed at the late stage of AD suggest a decrease in the efficiency of the recurrent GC-mediated inhibition of MCs. The interaction between MCs and GCs has been postulated to produce network oscillations in the OB that are associated with synchronous MC firing (Ravel et al., 2003; Beshel et al., 2007; Lagier et al., 2007). Impaired ultrastructural parameters of dendrodendritic synapses were always accompanied by abnormal synaptic transmission in APP/PS1 mice.

Synaptic transmission between dendrites is a major contributor to olfactory processing. In the OB, the glutamate released from MC dendrites excites the dendrites of GCs, and the excited dendrites mediates the GABAergic inhibition of MCs by releasing GABA (Lagier et al., 2007). GABA_A_Rs are ligand-gated Cl^−^ channels that mediate most of the fast inhibitory action of GABA in the central nervous system (CNS; Pallotto and Deprez, 2014). The affinity of the binding of GABA to GABA_A_Rs influence GABAergic synaptic transmission during olfaction processing. LFP oscillations in the mammalian OB represent coordinated neural activity that is dynamically regulated during olfactory processing. The γ rhythms strongly depend on the behavioral context and odor quality (Kay et al., 2009), and the γ oscillations in the OB are functionally related to the discrimination of overlapping odor input patterns (Beshel et al., 2007; Lepousez and Lledo, 2013). The γ oscillations reflect the synchronized spike discharges from MCs and local network activity (Schoppa, 2006; Sohal et al., 2009; Zhang et al., 2018). The γ oscillations rely on the dendrodendritic microcircuit between MCs and GCs and not on other synaptic interactions, such as gap-junction coupling or interneuron-interneuron connections (Lepousez and Lledo, 2013). The long-range synchronization between remote MCs operates selectively in the low-γ rhythms, whereas high-γ rhythms are spatially more restricted and represent the local dendrodendritic interactions (Lepousez and Lledo, 2013). At the early stage of AD, the γ oscillations in the EPL of APP/PS1 mice were significantly increased. Moreover, the older mice suffered serious olfactory dysfunction accompanied by an aberrant augmentation of γ oscillations in the EPL. The aberrantly increased γ oscillations in APP/PS1 mice indicated disrupted synchronized spike discharges from MCs, impaired synaptic transmission between GCs and MCs and abnormalities in olfactory processing in the OB. The application of a GABA_A_R agonist (muscimol) resulted in reduced γ oscillations, and a GABA_A_R antagonist (bicuculline) induced enhanced γ oscillations. The pharmacological treatments indicated that the regulation of the GC-mediated inhibition of MCs through modulation of the activities of GABA_A_Rs plays a key role in synaptic transmission between MCs and GCs and affects the γ oscillations. Moreover, a pharmacological decrease in the GC-mediated inhibition of MCs and an increase in the excitatory/inhibitory balance in MCs through the application of a GABA_A_R antagonist enhance the activity of MCs and long-range γ synchronization in a timely manner. Manipulations that aberrantly increase excitation/inhibition can reportedly impair odor mixture discrimination and slow the time required to discriminate between related odors (Lepousez and Lledo, 2013). Therefore, the enhanced γ oscillations in APP/PS1 mice might be a consequence of an increased excitatory/inhibitory balance in MCs due to a decreased GC-mediated inhibition of MCs. An increase in the GC-mediated inhibition of MCs and a decrease in the excitation/inhibition of MCs by GABA_A_R agonist (muscimol) could reduce the enhanced γ oscillations in APP/PS1 mice. The findings that pharmacologically treatment with a GABA_A_R agonist (muscimol) resulted in reduced γ oscillations support the hypothesis that altered GABAergic inhibition might underlie the aberrant γ oscillations in APP/PS1 mice.

MCs are necessary for generating spontaneous γ oscillations and for mediating the increased γ oscillations (Lepousez and Lledo, 2013). As the primary output neurons in the OB, MCs delivery the processed olfactory information in the OB to an advanced olfactory CNS. In the present study, the increased firing activity of MCs in APP/PS1 mice indicated abnormalities in olfactory information integration and transmission, which was consistent with olfactory disorder. The overexcitability of MCs was consistent with the neuronal hyperexcitability in higher-order cortical regions of AD patients and transgenic AD model mice and has been reported to be caused by Aβ (Wesson et al., 2011; Xu et al., 2015; Hu et al., 2017). At the early stage of AD, Aβ deposits mainly accumulated in MCs, although a small mount was detected within GCs. The decreased levels of synaptic vesicle-associated protein and the increased synaptic cleft of symmetrical synapses between GCs and MCs indicated aberrant synaptic transmission, which was consistent with the aberrant γ oscillations. In addition, the increased firing rates of MCs indicated the hyperexcitability of MCs and a failure of GC-mediated regulation. We hypothesized that the hyperexcitability of MCs and the observed aberrant synaptic transmission might contribute to the olfactory disorder observed early in AD. At the late stage of AD, excessive Aβ deposits accumulated in the OB, particularly within the GCs and MCs. In the OB, the dendrites of GCs, whose soma are located in the GCL, can regulate the activities of MCs through interaction with the dendrites of GCs. In addition, normal GC structure is essential for maintaining the normal activities of MCs. The decreased dendritic spine density of GCs in the presence of excessive Aβ deposits provides morphological evidence for a weakened inhibitory control of MCs by GCs. In addition, impaired structures of dendrodendritic synapses between GCs and MCs indicate damaged excitatory interactions between MCs and GCs and a reduced efficiency of inhibitory synapses to MCs. Aberrantly increased γ oscillations, which rely on the GC-mediated inhibition of MCs, verify the weakened GC-mediated inhibition of MCs (Lepousez and Lledo, 2013). Based on the morphological changes of GCs and dendrodendritic synapses, the MC hyperexcitability might be the result of Aβ toxicity-induced neurotoxicity within MCs and weakened inhibition of MCs by GCs. Based on the above-described results, we speculate that aberrant excitatory synaptic transmission and preserved inhibitory synaptic transmission might decrease the excitatory/inhibitory balance of MCs and enhance γ oscillations in APP/PS1 mice. In addition, the Aβ toxicity-induced MCs hyperexcitability signify aberrant olfactory information processing and transmission, which might contribute to olfactory dysfunction.

Taken together, our findings illustrated abnormal morphological changes in dendrodendritic synapses and disturbed local dendrodendritic neuronal circuits between GCs and MCs in the presence of excessive Aβ deposits, and these effects affected odorant processing and resulted in abnormal output signals from MCs. In addition, the impairments in the local inhibitory circuits and the excitatory/inhibitory balance lead to aberrantly enhanced γ oscillations, which might be responsible for the altered responses manifested as olfactory disorders in the AD mice. Our findings of local microcircuit impairments between GCs and MCs in the OB will help researchers obtain a better understanding of the synaptic mechanisms underlying early olfactory dysfunction in patients with AD. Additionally, the pharmacological and physiological manipulation of MC inhibition in patients with AD might provide a potential therapeutic strategy for this disease.

## Study Limitations and Future Directions

We verified the impaired structure and function of dendrodendritic synapses in the presence of excessive Aβ deposits and demonstrated that abnormal dendrodendritic inhibition in MCs might contribute to olfactory dysfunction. However, whether and how Aβ deposits induce impaired dendrodendritic inhibition of MCs from GCs and thereby lead to olfactory deficits in APP/PS1 mice require further investigation. In our preliminary study, pharmacological treatments with GABA_A_R agonist weakened the aberrantly increased γ oscillations. Therefore, we will utilize other methods in the future to strengthen the transmission of dendrodendritic inhibition from GCs onto MCs *in vivo* and the effects on olfactory behaviors in APP/PS1 mice.

## Conclusion

In summary, this study showed that APP/PS1 mice at sequential age stages exhibited gradual reductions in the dendritic spine density of GCs and continuous impairments in the synaptic interface parameters of the dendrodendritic synapses between GCs and MCs in the presence of excessive Aβ deposits. In addition, we observed aberrantly enhanced γ oscillations in the OB of APP/PS1 mice. Taken together, the data illustrate the structural and functional changes in the GC-mediated dendrodendritic inhibition of MCs in APP/PS1 mice and might help elucidate the mechanism of olfactory dysfunction in AD.

## Author Contributions

XY and SLin conceived and designed the study. LS, JW, XW, JL and CT acquired the data and prepared the figures. WL and SLi analyzed the data and wrote the manuscript.

## Conflict of Interest Statement

The authors declare that the research was conducted in the absence of any commercial or financial relationships that could be construed as a potential conflict of interest.
